# MEX3C as a potential target for hepatocellular carcinoma drug and immunity: combined therapy with Lenvatinib

**DOI:** 10.1186/s12885-023-11320-4

**Published:** 2023-10-12

**Authors:** Jinhui Guo, Jie Zhao, Qiuran Xu, Dongsheng Huang

**Affiliations:** 1 Qingdao Medical College, Qingdao University, Qingdao, China; 2 Key Laboratory of Tumor Molecular Diagnosis and Individualized Medicine of Zhejiang Province, Zhejiang Provincial People’s Hospital, Affiliated People’s Hospital, Hangzhou Medical College, Hangzhou, Zhejiang 310014 China; 3 College of Biotechnology and Bioengineering, Zhejiang University of Technology, Hangzhou, China

**Keywords:** MEX3C, Lenvatinib, Immune, Microenvironment, Hepatocellular carcinoma

## Abstract

**Background:**

The immune microenvironment within hepatocellular carcinoma (HCC) is remarkably intricate. Although the combination of an immune checkpoint inhibitor and Lenvatinib can extend the overall survival of HCC patients, the outcome remains suboptimal.

**Methods:**

We assessed alterations in MEX3C expression during hepatocarcinogenesis by validating multiple databases and subsequently developed a predictive model. Subsequently, we enriched the associated genes of MEX3C to investigate its functional role. We examined the correlation between MEX3C expression levels and immune infiltrating cells. The effects of MEX3C knockdown and Lenvatinib on hepatoma cells were observed by cell function experiments.

**Results:**

MEX3C expression is elevated in HCC compared to normal tissues, and its high expression correlates with poor prognosis. Immune checkpoint expression was elevated in the high MEX3C expression group, concomitant with heightened myeloid-derived suppressor cell (MDSC) expression. The combination of MEX3C knockdown and Lenvatinib demonstrated a stronger inhibitory effect on HCC cells compared to Lenvatinib alone.

**Conclusion:**

MEX3C shows promise as a potential therapeutic target for treating HCC. Furthermore, the combination of MEX3C knockdown and Lenvatinib could offer a novel therapeutic avenue for HCC treatment.

**Supplementary Information:**

The online version contains supplementary material available at 10.1186/s12885-023-11320-4.

## Background

Liver cancer is among the prevailing clinical malignancies, and its global incidence has escalated in recent years, positioning it as the fourth foremost contributor to global cancer-related mortality [1]. Hepatocellular carcinoma (HCC) is the most common type of liver cancer, accounting for approximately 85% of all primary liver cancer cases [2]. Early detection of HCC is challenging, often leading to diagnosis at advanced stages that preclude the opportunity for curative surgery [3, 4]. For more than a decade, Sorafenib has become the only first-line targeted drug approved for advanced HCC [5, 6]. In 2018, clinical trials demonstrated that Lenvatinib’s effectiveness in treating advanced HCC was non-inferior to that of sorafenib, leading to its designation as a first-line option for targeted therapy in advanced HCC [7, 8]. Furthermore, Lenvatinib demonstrated significantly superior rates of progression-free survival and objective remission compared to Sorafenib [9]. Lenvatinib inhibits tyrosine kinase and exerts tumor suppression, and the main targets are rearranged during transfection (RET), vascular endothelial growth factor receptor (VEGFR1-3), platelet-derived growth factor receptor α (PDGFRα), fibroblast growth factor receptor (FGFR1-4), and KIT. In addition, Lenvatinib has immunomodulatory activity, showing stronger antitumor activity in immunocompetent mice [10].

In recent years, cancer immunotherapy has achieved tremendous clinical progress in solid tumors [11]. The immunotherapy response rate in HCC was merely 15%, with treatment failure potentially stemming from the immunosuppressive nature of the HCC microenvironment [12, 13]. Studies have shown that Lenvatinib can affect the immune microenvironment and increase the percentage of CD8 + T cells in HCC [14, 15]. Lenvatinib could block FGFR4, resulting in decreased tumor PD-L1 expression, providing a rationale for combined immunotherapy regimens [16]. The objective effective rate of Lenvatinib combined with Pembrolizumab was 44.8%, showing strong anti-tumor activity [17]. Nonetheless, a subset of patients exhibits resistance to the combined regimen of immunotherapy and Lenvatinib. Therefore, it is still necessary to find new targets to improve the combination therapy scheme.

MEX3C is an RNA binding protein that may affect the energy balance of cells [18]. MEX3C has been found in extracellular secretions, but its overall function is still unclear [19]. In this article, we elucidated the biological function of MEX3C in HCC and further explored the significance of MEX3C expression on patients’ prognosis and the immune microenvironment. Finally, we verified the combined effect of MEX3C and Lenvatinib on hepatoma cells through functional experiments. Our findings have offered valuable insights for the investigation of innovative combined immunotherapy strategies for future HCC studies.

## Methods

### Patients and samples

RNA sequencing data and clinical information from both normal liver tissues and HCC tissues were obtained from The Cancer Genome Atlas (TCGA) database. RNA sequencing data for validating the differential gene expression between normal tissues and HCC tissues were obtained from the Gene Expression Omnibus (GEO) database (GSE14520_GPL571 and GSE76427_GPL10558). Clinical and follow-up information utilized for constructing the prediction model was obtained from the International Cancer Genomics Consortium (ICGC LIRI-JP) website.

### Prognostic analysis

Using the average MEX3C expression value as a threshold, the samples were categorized into high and low expression groups. Subsequently, survival analysis was conducted utilizing the R package (survival). Univariate analysis was performed first, and factors with p < 0.05 were included in multivariate cox retrospective analysis.

Predictive prognosis models were constructed using the R(rms) package. The accuracy of survival predictions was assessed using calibration curves and C-index values.

### Immune analysis

ESTIMATE can assess the tumor microenvironment in tumor samples based on expression data. The R package (estimate) was utilized to estimate the stromal and immune cell scores (Stromal Score, Immune Score, Estimate Score, and Tumor Purity) for HCC patients.

The R package (e1071, parallel, preprocessCore) was employed to estimate the proportion of immune cells in a sample using the CIBERSORT method. The outcome was computed by CIBERSORT, representing the proportions of 22 distinct immune cell types.

The ssGSEA algorithm was primarily executed using the R package (gsva), enabling the quantification of immune infiltration in HCC. The obtained results represented the degree of infiltration of the 28 immune cell types within the sample.

MEX3C was categorized into two groups based on the average expression level, followed by a comparison of expression differences in immune checkpoint genes (PD1, PDL1, and PDL2) between these groups.

### Functional enrichment analysis

Initially, Pearson correlation analysis was conducted on tumor expression data to identify genes associated with MEX3C. Subsequently, the relevant gene data were ranked based on their correlation (cor) value and P value. The top 500 genes were then selected for enrichment analysis, including Gene Ontology (GO) analysis, Kyoto Encyclopedia of Genes and Genomes (KEGG) analysis, and Gene Set Enrichment Analysis (GSEA) analysis. These analyses were carried out using the R package (clusterProfiler) [20].

### Co-expression network analysis

We constructed co-expression networks using Differentially Expressed Genes (|log2(fold-change)|> 1, p < 0.05) data. Modules were linked to immunity using the R package (WGCNA), while gene significance (GS) and module membership (MM) were calculated [21]. The genes with GS > 0.2 and MM > 0.8 in the black module were defined as hub genes, and correlation analysis was performed. A protein-protein interaction (PPI) network was constructed with the STRING database.

### Cell counting Kit-8 assay

A total of 2 × 10^3^ cells were seeded per well in 96-well plates, followed by cell adhesion to the surface and treatment with a gradient of drugs (0–20µM). Following 72 h of drug exposure, Cell Counting Kit-8 reagent (Yeasen Biotechnology, 40203ES80) was diluted at a 1:10 ratio and added to each well. The samples were then incubated at 37 °C for a duration of two hours. Finally, the absorbance of the cells was measured at a wavelength of 450 nm [22].

### Wounding healing assay

The scratch chamber (Ibidi, 80,209) was positioned on a 12-well plate, and 7 × 10^5^ cells were inoculated on both sides of the scratch chamber. After the cells adhered to the wall, the scratch chamber was removed, and the cells were placed in a low serum medium. Subsequently, Lenvatinib drug was added to the respective wells. Finally, images were captured at 0-hour and 24-hour time points to compare the cellular healing rates among the different groups.

### Transwell migration assay

The chambers (Corning, 3464) were positioned in 24-well plates, and 200 µL of serum-free cell suspension containing 2 × 10^4^ cells was added to each upper chamber. Each lower chamber was filled with 600 µL of 10% serum medium. Following a 24-hour incubation at 37 °C, cells were fixed with methanol and subsequently stained with crystal violet dye. Cells in the upper chamber were gently wiped, and images were captured and recorded for the cells that migrated across the chamber’s transmembrane.

### RNA extraction and fluorescence quantitative PCR

RNA extraction from cells was performed using the RNA rapid extraction kit (ESscience Biotech, RN001). Complementary DNA (cDNA) was synthesized from the extracted RNAs using the kit (Takara, RR037A-1). Real-time fluorescent PCR quantification was performed using the SYBR Green reagent (Yeasen, 11184ES08). The primer sequences can be found in **Supplementary Table 1**.

### Western blotting

The supernatant protein was collected following the lysis of Huh7 cells. Protein samples were quantified using a BCA Kit. Protein samples were separated using sodium dodecyl sulfate polyacrylamide gel electrophoresis (SDS-PAGE) and transferred to polyvinylidene difluoride membranes (PVDF). At the end of transfer, blocking was performed using 5% nonfat milk for about 1 h at room temperature conditions. Blots were probed with anti-MEX3C (Santa, 398,440) and anti-β-Actin (Affinity, AF7018) antibodies, which were incubated overnight at 4 ° C on a shaker. The next day, the membranes were washed using Tris-buffered saline with Tween 20 (TBST). Subsequently, the membrane was next washed using TBST after binding with secondary antibodies for 1 h at room temperature. Finally, the amount of protein expression in the sample bands was detected using chemiluminescence.

### Transfected SIRNA

5 × 10^5^ cells were inoculated in a six well culture plate and transfected when the cell density reached 70%. Lipo3000 was used as transfection reagent. The siRNA of MEX3C (GenePharma, Shanghai, China) was added to the medium and transfected for about 48 h. After two days of culture, it was used for subsequent experiments. The primer sequences can be found in **Supplementary Table 2**.

### Statistical analysis

Student’s t-test was used to analyze the difference between the means of the two groups. All data were statistically analyzed using R (v4.2.0), SPSS (v25.0) and GraphPad Prism (8.0).

## Results

### Compared with normal liver tissue, MEX3C was enriched in HCC

**Fig. 1:**
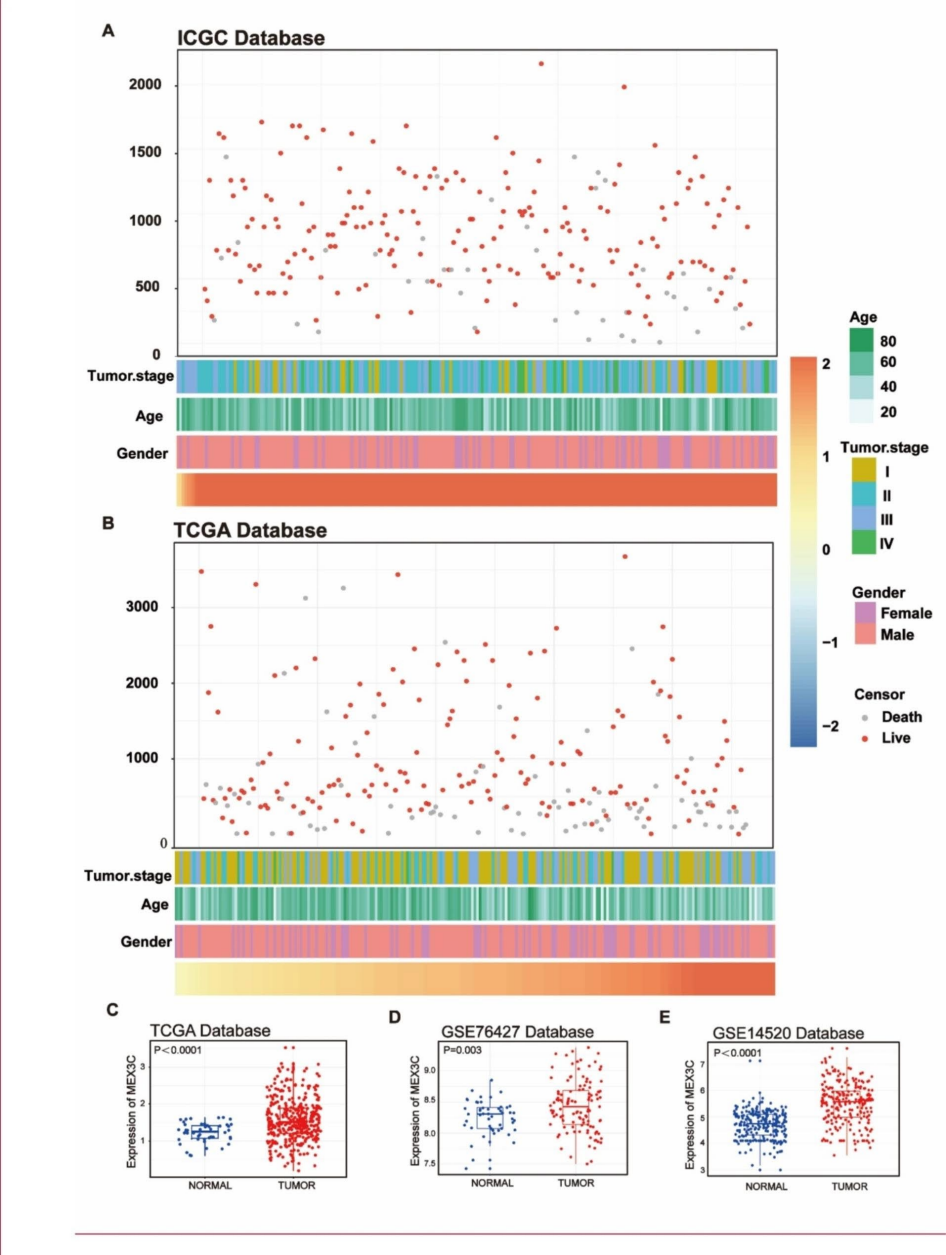
Correlation between MEX3C expression and clinicopathological characteristics of HCC. **(A–B)** Description of the relationship between MEX3C and clinical features. **(C-E)** Comparison of MEX3C in tumor tissue and normal tissue

(Fig. 1A-B) showed the distribution of clinicopathological features and prognosis with increasing MEX3C expression levels in tumors in ICGC and TCGA database. In TCGA database, we found that the expression of MEX3C mRNA in HCC was higher than that in normal liver tissue (Fig. 1C). Consistent findings were observed in both GSE76427 and GSE14520 databases (Fig. 1D-E). The results suggested that MEX3C may be overexpressed in HCC, thereby suggesting its viability as a therapeutic target in HCC treatment.

### The expression of MEX3C was correlated with overall survival and stood as an independent prognostic factor among patients with HCC

**Fig. 2:**
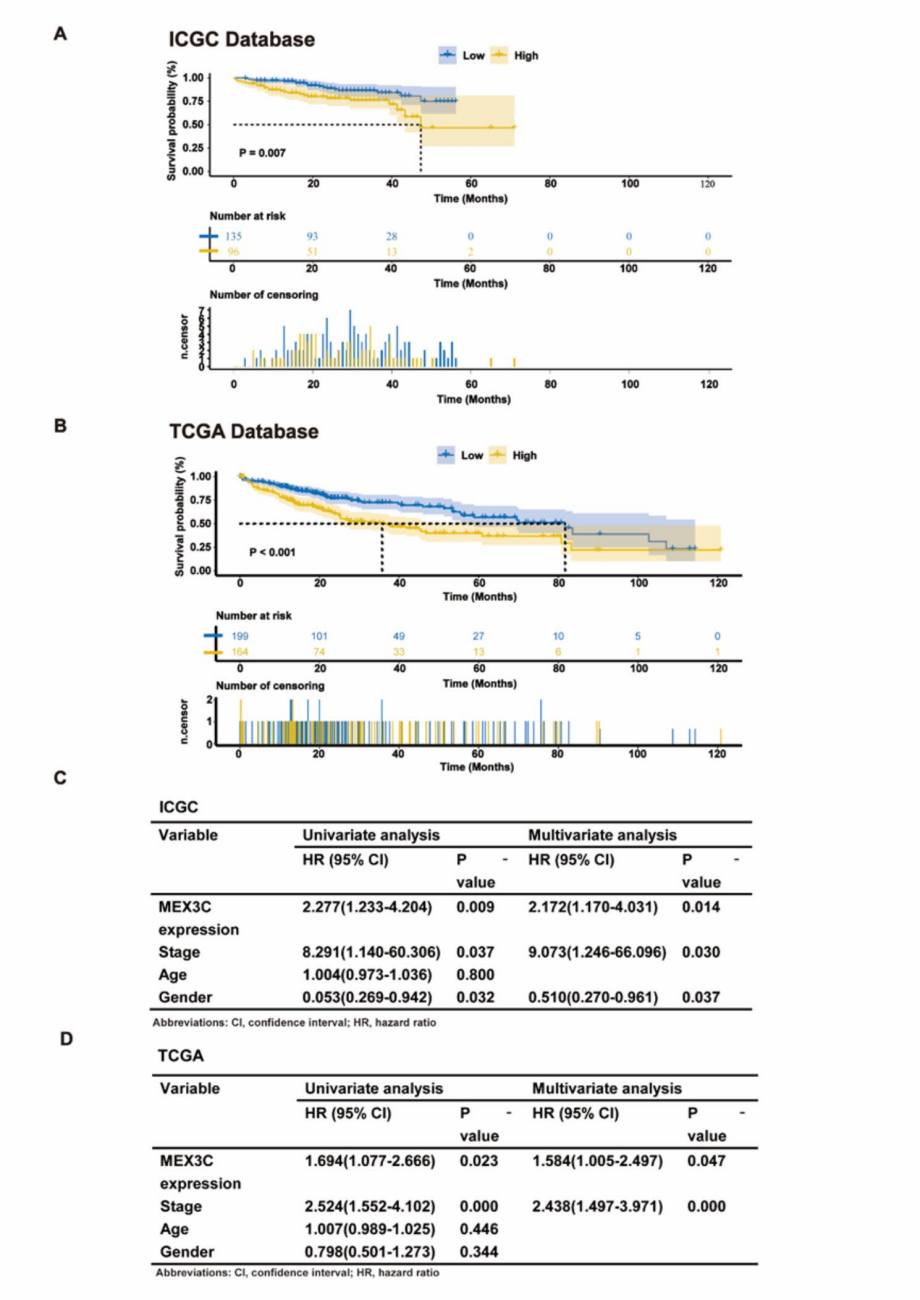
Prognostic significance of MEX3C in HCC. **(A–B)** Kaplan-Meier overall survival of MEX3C in the ICGC and the TCGA. **(C–D)** OS: Univariate and Multivariate Cox Regression Analysis in the TCGA and the ICGC

Next, we assessed the prognostic significance of MEX3C in patients with HCC. Kaplan-Meier curve showed that patients with HCC with higher MEX3C expression had lower overall survival (OS), which was statistically significant (p < 0.05) (Fig. 2A). The correlation between high expression of MEX3C and OS was verified in the TCGA database **(**Fig. 2B**)**. Univariate Cox analysis showed that MEX3C expression (HR: 2.211, 95% CI: 1.233–4.204), gender (HR: 0.053, 95% CI: 0.269–0.942) and tumor stage (HR: 8.291, 95% CI: 1.140-60.306) were independent prognostic factors for HCC patients (Fig. 2C). Incorporating the above factors into cox multivariate analysis showed that MEX3C was still an independent prognostic risk factor affecting survival (p < 0.05). In the cox retrospective analysis of the TCGA database, MEX3C was not affected by tumor stage and was an independent prognostic factor (Fig. 2D). These findings implied a correlation between MEX3C overexpression and an unfavorable prognosis.

### Construction of a nomogram based on mex3c expression

**Fig. 3:**
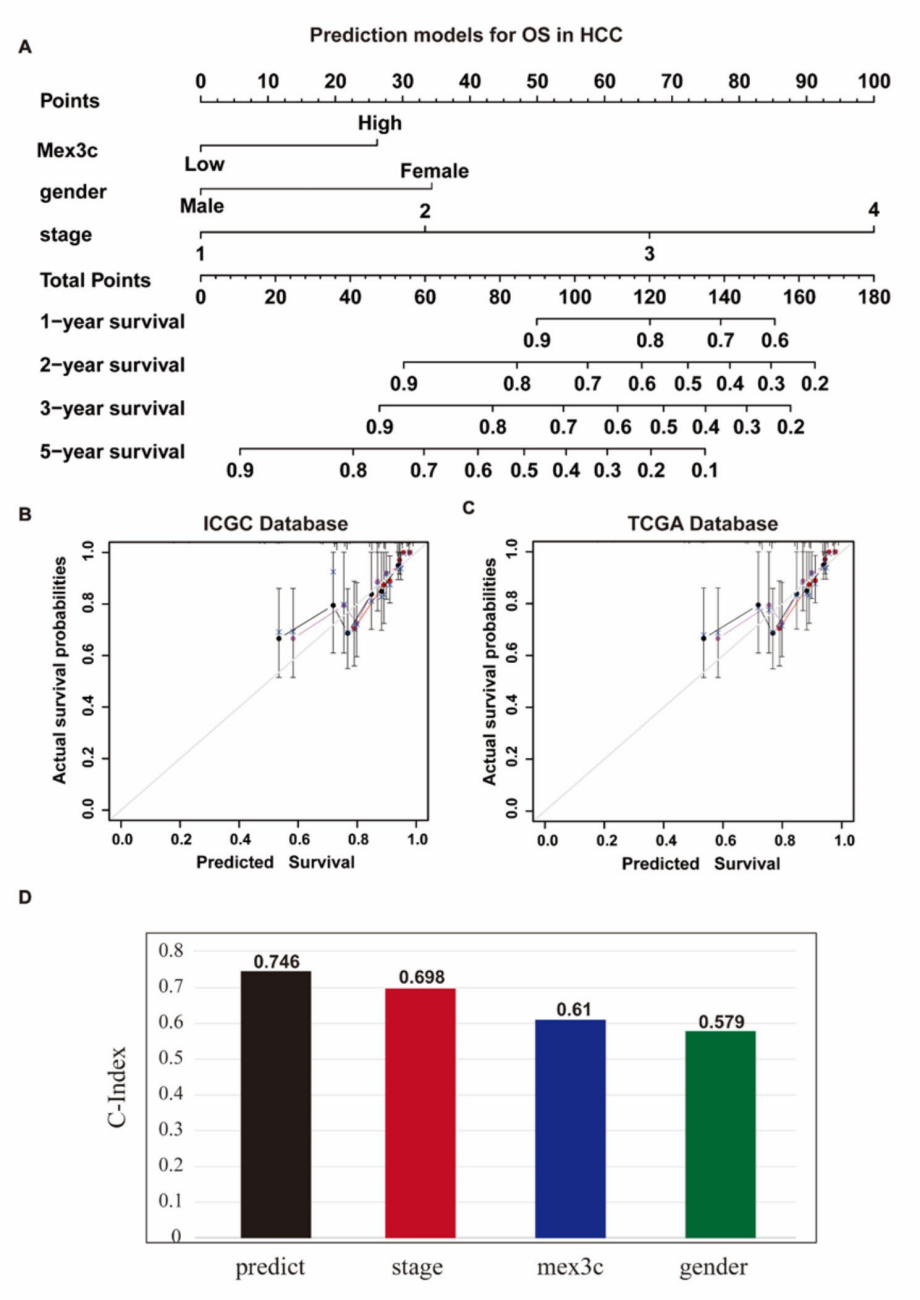
Construction of a prediction model of MEX3C in HCC. **(A)** MEX3C is combined with clinicopathological features to predict prognosis. **(B–C)** Calibration curves of ICGC training database and TCGA validation database. **(D)** Predictive effect of ME3C, clinicopathological features, and predictive models

To apply the prognostic significance of MEX3C to the clinic, we constructed a nomogram model. We integrated clinicopathological factors (gender and tumor stage) and MEX3C expression levels to predict 1-, 2-, 3-, and 5-year survival probabilities of patients in the clinic (Fig. 3A). The C-index of this nomogram model was 0.746, which was superior to the predictive model with tumor stage as a single factor (Fig. 3D). The actual values ​​in the ICGC database show good agreement in the calibration curve (Fig. 3B). The calibration curve of the TCGA database is the validation set, which verifies the accuracy of the prediction (Fig. 3C). These results suggest that MEX3C, as a prognostic marker for HCC, can improve the prediction accuracy of tumor staging for survival.

### Go analysis, GSEA analysis and KEEG pathway analysis on MEX3C

**Fig. 4:**
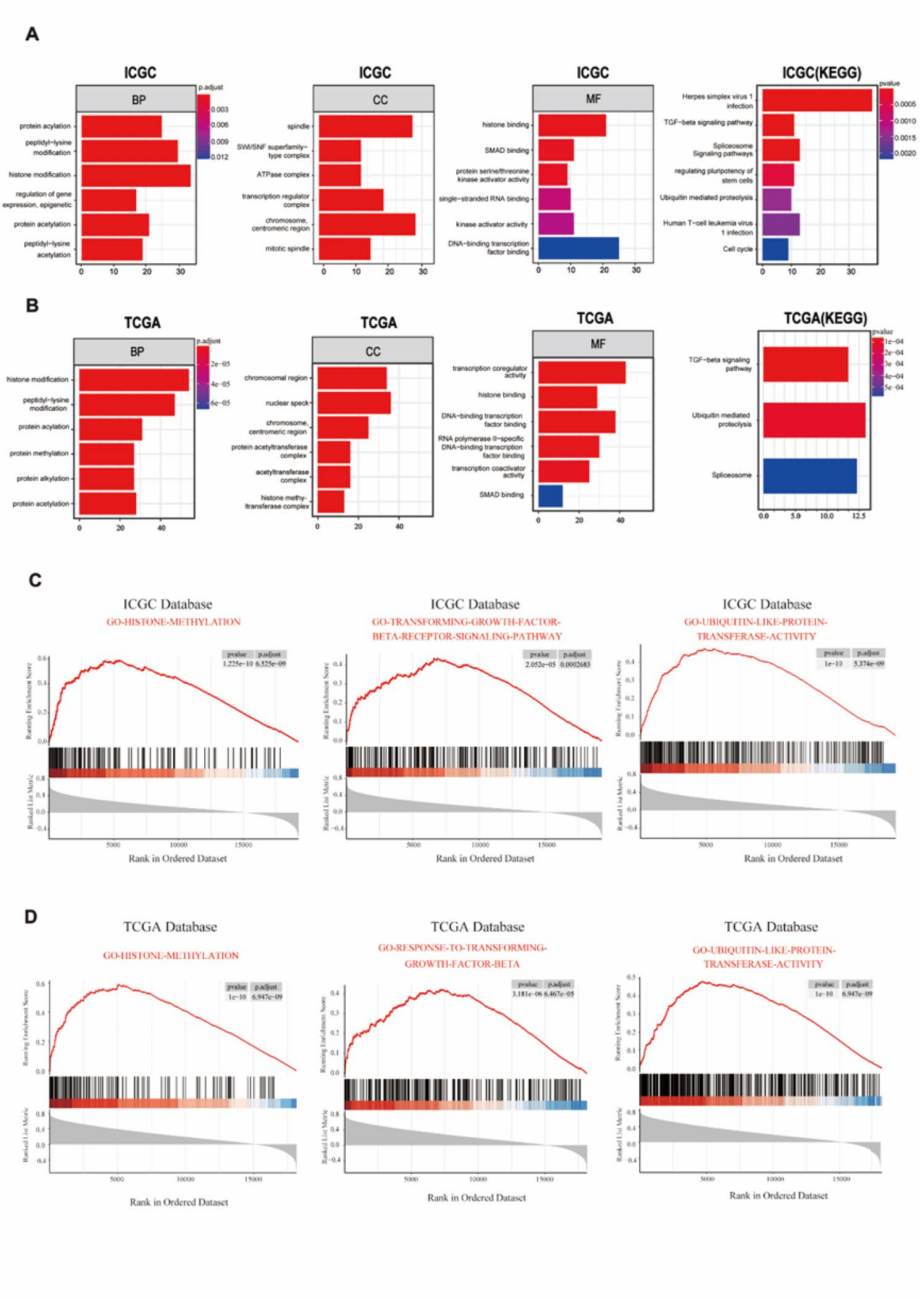
Enrichment analysis of GO, KEGG and GSVA. **(A-B)** BP, CC, MF and KEGG pathway analysis of MEX3C in the ICGC and the TCGA database. **(C-D)** Analysis of MEX3C in the ICGC and the TCGA database

To gain insight into the biological functions of MEX3C, we performed GO analysis and KEGG pathway enrichment analysis on genes associated with MEX3C. The top 6 enrichment items of the ICGC and TCGA analysis results were taken respectively, and then the overlapping enrichment items of the two databases were analyzed. GO-BP analysis shows that the overlapping enrichment items of the two databases are histone modification, peptidyl-lysine modification, protein acylation and protein acetylation (Fig. 4A-B). For GO-CC analysis, the overlapping enrichment items are chromosome, centromeric region (Fig. 4A-B). GO-MF analysis shows that the overlapping enrichment items are histone binding, DNA-binding transfer factor binding and SMAD binding (Fig. 4A-B). The main pathways revealed by the KEGG results were transforming Growth Factor-Beta (TGF-β) signaling pathway and Ubiquitin mediated proteolysis. Finally, GSEA analysis also suggested that MEX3C may play a role in histone, TGF-β and ubiquitination (Fig. 4C-D).

### Expression of MEX3C and immune cell infiltration

**Fig. 5:**
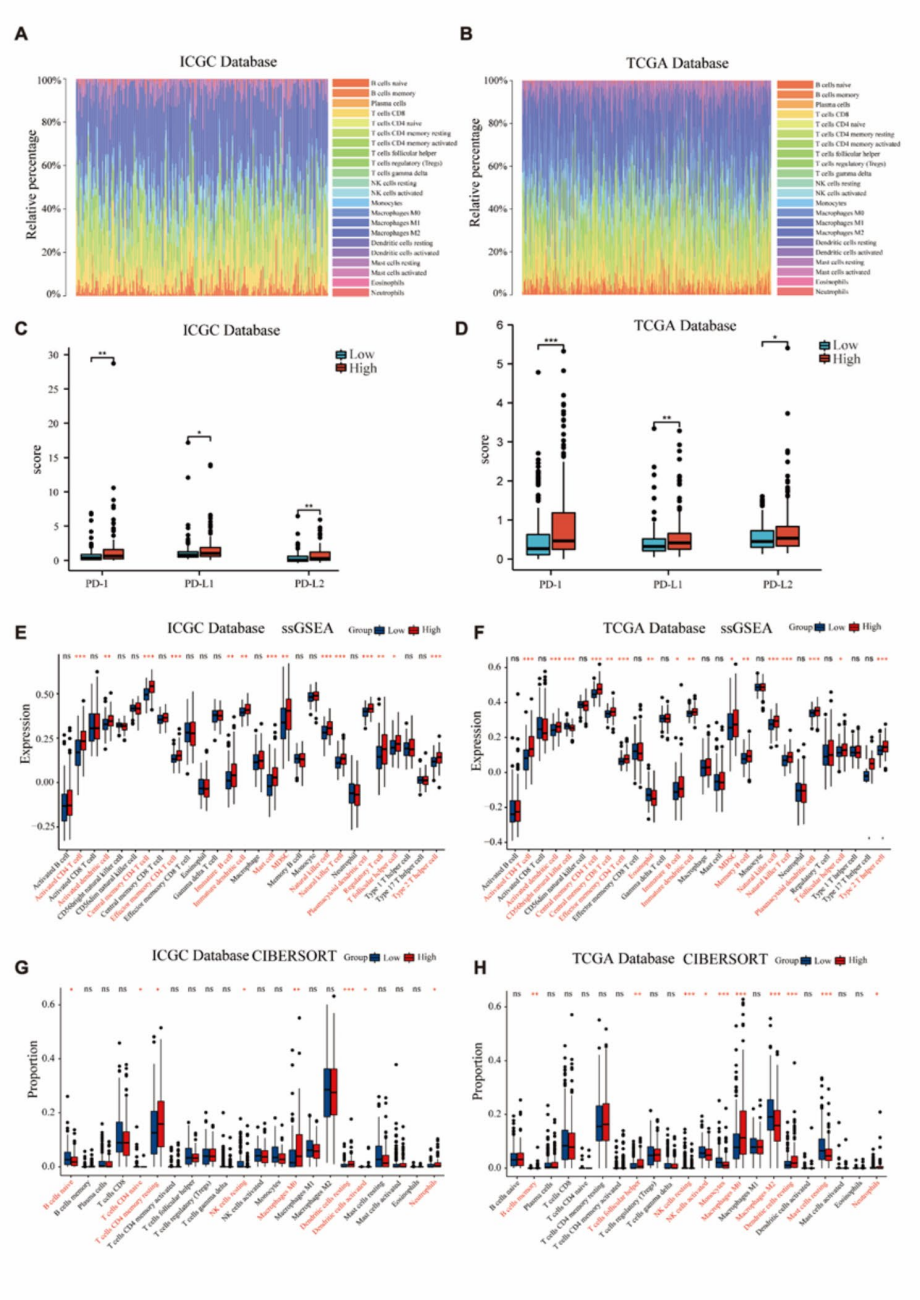
Significance of MEX3C in the tumor immune microenvironment of HCC. **(A-B)** The heatmap shows the proportion of immune cells in each patient’s HCC in ICGC and TCGA databases. **(C-D)** Differences in immune checkpoints (PD1, PDL1, and PDL2) between MEX3C high- and low-expression groups. **(E-F)** Difference of immune cell expression between MEX3C high expression group and low expression group. **(G-H)** Differences in proportion of immune cells between MEX3C overexpression group and low expression group

(Fig. 5A-B) showed the proportion of immune cells in HCC from two databases. We then assessed the relationship between the level of MEX3C expression and immune checkpoints, and the immune targets (PD1, PDL1 and PDL2) of the MEX3C high expression group were also elevated (Fig. 5C-D). The analysis of CIBERSORT and ssGSEA showed that the level of MEX3C expression was correlated with the number and proportion of some immune infiltrating cells in the HCC microenvironment (Fig. 5E-H). The ssGSEA of the ICGC database showed that the expression levels of MDSCs and Regulatory T cells (Tregs) in the tumor microenvironment of the MEX3C high expression group increased (Fig. 5E). The ssGSEA of the TCGA database showed that the expression of MDSCs in the tumor microenvironment of the MEX3C high expression group increased, while the expression of CD56bright natural killer cells decreased (Fig. 5F). The CIBERSORT results of the TCGA database showed that the proportion of activated natural killer cells in the tumor microenvironment of the MEX3C high expression group was reduced (Fig. 5H).

### Differentially expressed genes and tumor microenvironment

**Fig. 6:**
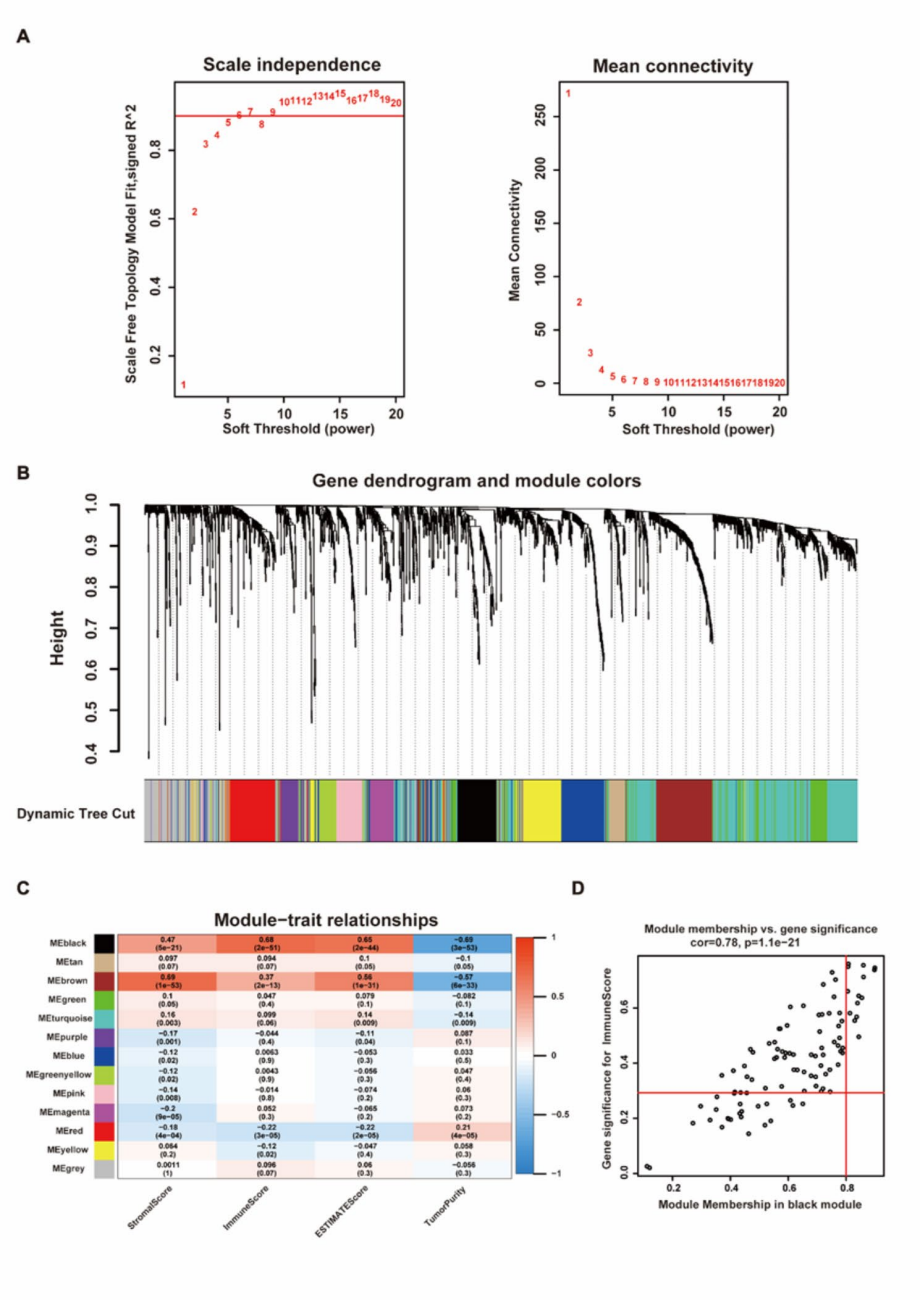
Module analysis related to immune characteristics of HCC. **(A)** Analysis of soft-thresholding power in WGCNA. **(B)** Gene dendrogram and module colors. **(C)** Heatmap of the correlation between the module eigengenes and tumor microenvironment. **(D)** Module membership in the black module

We calculated the optimal soft threshold and started building the co-expression network (Fig. 6A). We use differentially expressed genes (|log2(fold-change)|> 1, p < 0.05) for clustering and get Dendrogram (Fig. 6B). An association analysis between modules and traits was performed to identify immune-related modules. (Fig. 6C). We found that the black module has a high correlation with immunity, and then extracted the hub genes in the black module for subsequent analysis (cor = 0.68, p = 2e-51). According to MM > 0.8 and GS > 0.25, we obtained 14 hub genes from the black module (IL21R, PTPN22, TFEC, PTPRC, WDFY4, GCSAM, P2RY10, GPR174, ITGA4, CCR4, GPR141, AIM2, TLR8, ADAMDEC1) (Fig. 6D).

### Correlation between hub genes and immunity

**Fig. 7:**
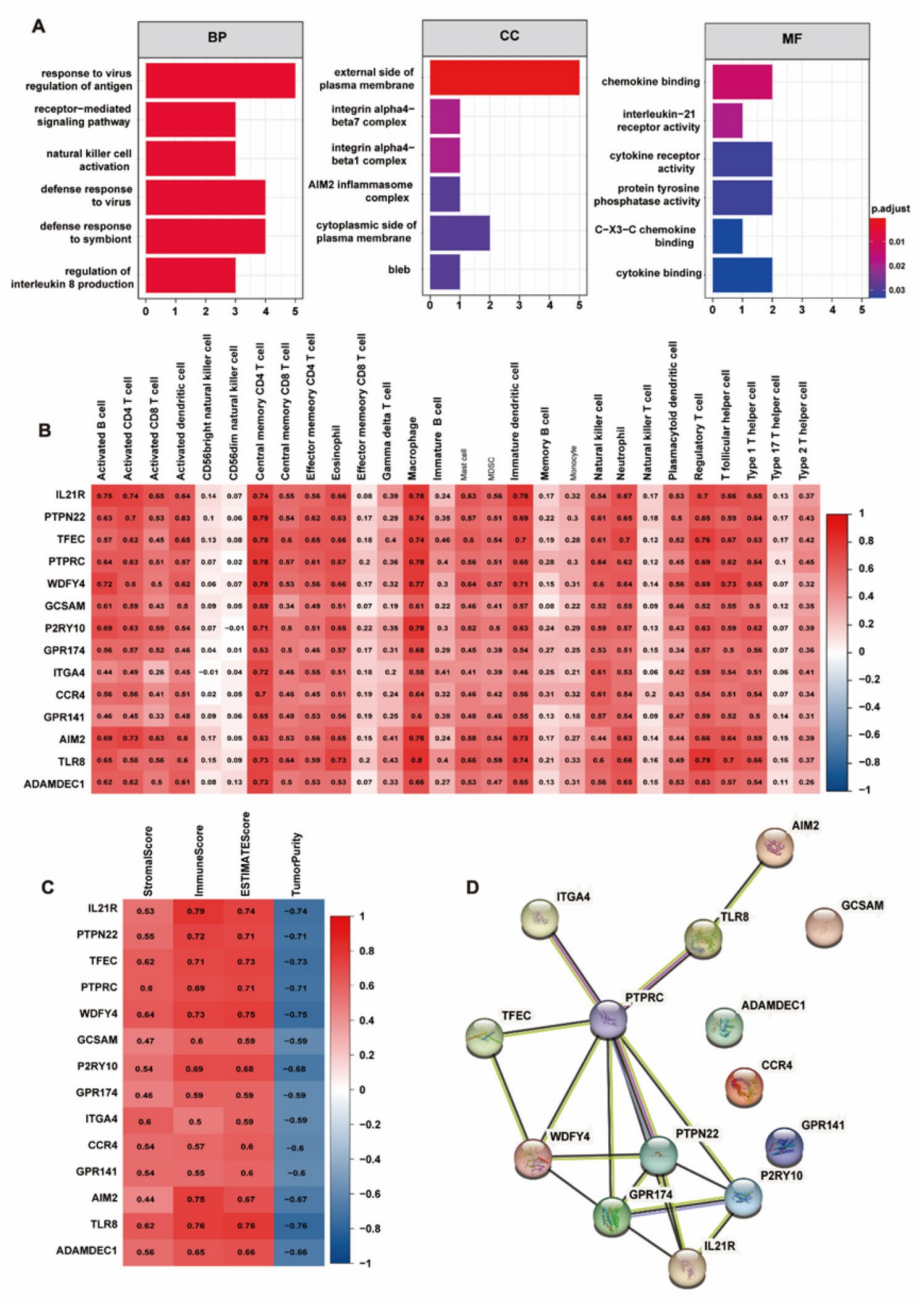
Analysis of hub genes in black module. **(A)** BP, CC and MF analysis of hub genes. **(B)** Correlation between hub genes and ssGSEA. **(C)** Correlation between hub genes and tumor microenvironment. **(D)** Protein interaction network of hub genes

In order to verify the relationship between genes and immunity in the black module, we first performed GO enrichment analysis on 14 hub genes, and the results showed that hub genes can affect the immune system (Fig. 7A). Next, we analyzed the correlation between hub genes and immune infiltration and tumor microenvironment, and the results showed that hub genes were closely related to immunity (Fig. 7B-C). Finally, the PPI network was constructed for the 14 hub genes, illustrating their interrelationships (Fig. 7D).

### Effects of knockdown of MEX3C and Lenvatinib on HUH7 cells

**Fig. 8:**
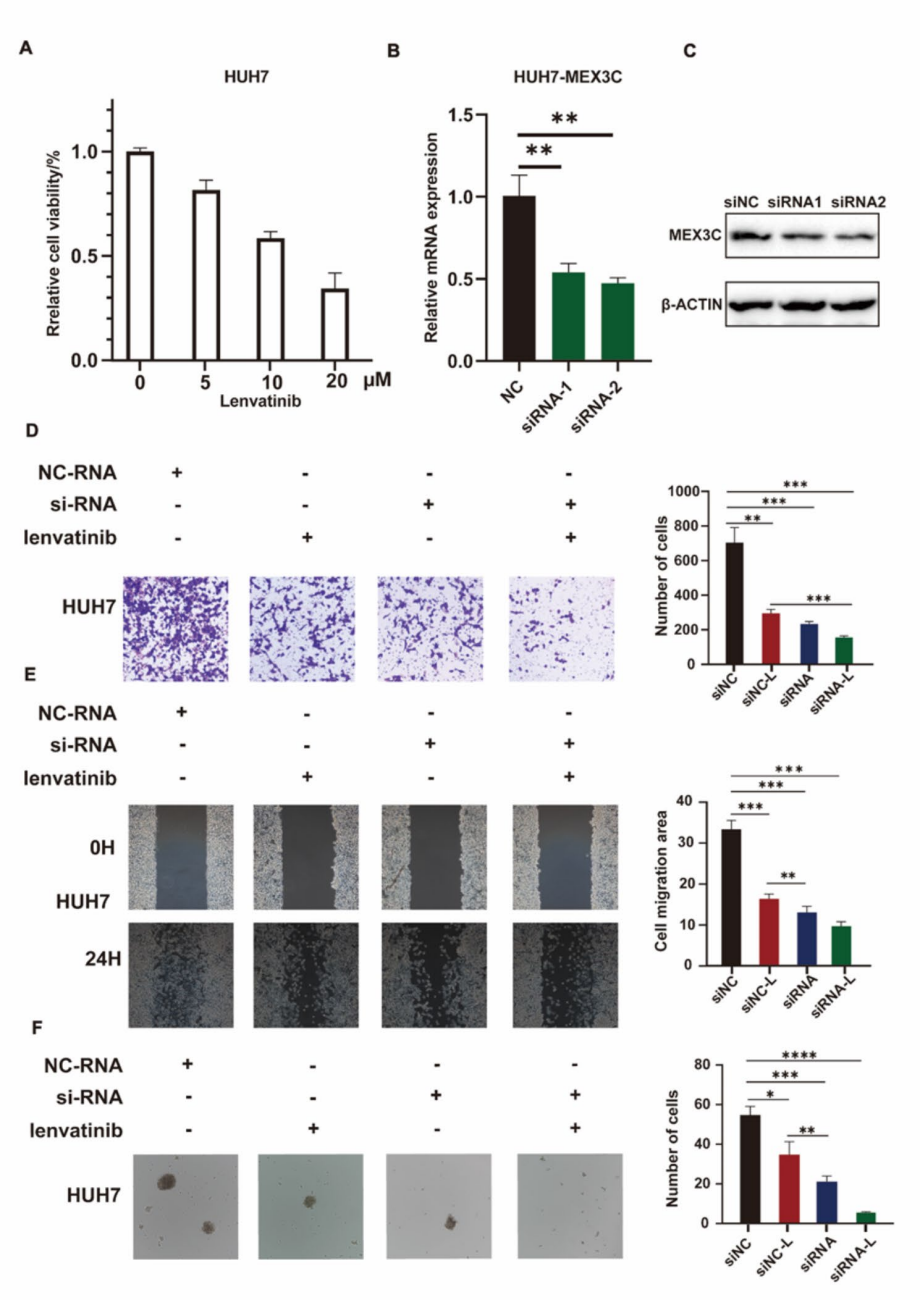
Combination of knockdown MEX3C and Lenvatinib in the treatment of HCC. **(A)** Determination of IC50 of Lenvatinib on HUH7 cells. **(B-C)** RT-PCR and western blot were used to verify the knock down efficiency of MEX3C. **(D)** Transwell migration assay was used to detect the invasion of HUH7 cells. **(E)** Wound healing assay was used to detect the invasion of HUH7 cells. **(F)** Sphere formation was used to detect the HUH7 cells stemness

In order to determine the effect of combination therapy of knockdown MEX3C and Lenvatinib on HCC, we conducted a series of functional experiments to verify it. We first performed cell viability assays to determine the IC50 values ​​of Lenvatinib in HUH7 cells (IC50 = 10.26) (Fig. 8A). Then we used siRNA targeting MEX3C to transfect HUH7 cells, and verified the knockout efficiency at the protein level and gene level (Fig. 8B-C). When compared to Lenvatinib treatment alone, the knockdown of MEX3C resulted in a significant reduction in both the invasive and migratory abilities of HUH7 cells. (Fig. 8D-E). To further verify the antiproliferative effects of Lenvatinib and knockdown of MEX3C in HUH7 cells, sphere formation experiments were also performed (Fig. 8F). The experimental results propose a feasible strategy involving the simultaneous targeting of MEX3C and administration of Lenvatinib.

## Discussion

In our study, MEX3C can independently affect the prognosis of patients, whether in univariate analysis or multivariate analysis. A noteworthy correlation was observed between elevated MEX3C gene expression and unfavorable prognosis. This discovery implies that targeting MEX3C could hold promise as a potential therapeutic strategy to enhance the prognosis for individuals diagnosed with HCC. Considering the large sample size of ICGC and TCGA databases, we built a prediction model for prognosis, and found that MEX3C can improve the prediction effectiveness of the model. The transwell and sphere formation assays indicated that MEX3C might significantly contribute to suppressing the invasion and stemness of HCC cells. This discovery offers a potential therapeutic avenue for clinical targeted therapy.

Lenvatinib-based combination therapy represents a significant recent breakthrough in HCC treatment, showing ongoing advancements. The combination of TACE and Lenvatinib enhances the objective response rate, outperforming transarterial chemoembolization (TACE) monotherapy [23]. In recent times, the synergistic application of Lenvatinib and an immune checkpoint inhibitor has yielded substantial outcomes, thus expanding the potential horizons for addressing HCC. However, Lenvatinib resistance is an Important obstacle to maintaining long-term therapeutic effects, and better targets are needed to enhance the killing effect of Lenvatinib. Subsequently, we intend to establish drug-resistant Lenvatinib cell lines and explore the potential impact of MEX3C on the resistance of HCC cells to Lenvatinib.

HCC contains a large number of immune cells, which provide a complex microenvironment for tumorigenesis and development [13]. Immune checkpoint inhibitors have played a solid role in the treatment of some cancers. Overexpression of immune checkpoint (PD1, PD-L1 and PD-L2) can lead to tumor immune escape [24, 25]. In the high expression group of MEX3C within HCC tissue, there was a statistically significant increase in the expression of immune checkpoints. The ssGSEA analysis revealed that elevated MEX3C expression is linked to a higher count of MDSCs in both databases. MDSCs have the ability to prompt the differentiation of regulatory T cells and facilitate the development of an immunosuppressive microenvironment surrounding cancer cells [26]. The accumulation of mononuclear MDSCs (M-MDSCs) is associated with heightened tumorigenicity in a mouse model of liver cancer [27]. Targeting the MEX3C gene could potentially influence immunosuppressive cells in the tumor microenvironment, including regulatory T cells and MDSCs, potentially enhancing the efficacy of immunotherapy.

Our analysis of pathways involving MEX3C-related genes revealed an enrichment of MEX3C within the TGF-β signaling pathway. The TGF-β signaling pathway assumes a pivotal role in both fibrosis and immune regulation within the HCC microenvironment [28]. TGF-β signaling can be exploited by cancer cells to reshape the immune microenvironment and foster the development of “immune evasion.“ Upon stimulation by TGF-β factor, the tumor microenvironment becomes abundant in collagen fibers, leading to the exclusion of T cells by fibroblasts at the tumor periphery. Concurrent inhibition of PDL1 and TGF-β in clinical contexts heightens tumor responsiveness to immunotherapy, resulting in diminished tumor volume [29]. Despite these findings, our study exhibits certain limitations, necessitating additional in vivo experiments for the substantiation of our conclusions. The absence of co-culture experiments to assess the impact of MEX3C on immune infiltration is notable. Additionally, due to the nature of the database, protein-level data is unavailable.

## Conclusions

Overall, our study encompassed an exhaustive exploration of MEX3C’s biological functionality, elucidating its potential utility as a prognostic marker for patients as well as its immunomodulatory influence. MEX3C exhibits associations with various immunosuppressive pathways, including TGF-β, MDSCs, and Tregs, while also augmenting the tumor-suppressive impact of Lenvatinib. These findings introduce a novel avenue for prospective immune combination strategies.

### Electronic supplementary material

Below is the link to the electronic supplementary material.

Supplementary Material 1

Supplementary Material 2

## Data Availability

All databases can be obtained from the public database: TCGA (https://portal.gdc.cancer.gov/ LIHC), ICGC (http://dcc.icgc.org LIRI-JP), and GEO (https://www.ncbi.nlm.nih.gov/geo/ GSE14520_GPL571 and GSE76427_GPL10558).

## Declarations

### Hepatoma cell

HUH7 are bought from the Chinese Academy of Sciences cell library (https://www.cellbank.org.cn/index.php).

## Abbreviations

HCC: Hepatocellular carcinoma
TCGA: The Cancer Genome Atlas
GEO: Gene Expression Ominibus
ICGC: International Cancer Genomics Consortium
KEGG: Kyoto Encyclopedia of Genes and Genomes
GO: Gene Ontology
GSEA: Gene Sets Enrichment Analysis
