# Supplementary material for: MEX3C as a potential target for hepatocellular carcinoma drug and immunity: combined therapy with Lenvatinib
Source: BMC Cancer. 2023 Oct 12;23:967. doi: 10.1186/s12885-023-11320-4 (PMC10568896; doi:10.1186/s12885-023-11320-4)
Supplement: Supplementary file 1 — Supplementary Material 1 [file 12885_2023_11320_MOESM1_ESM.docx]

# Supplementary Tables

**Table 1. Primers used for quantitative real-time PCR (RT-qPCR)**

| **Genes** | **Forward primer (5’-3’)** | **Reverse primer (5’-3’)** |
| --- | --- | --- |
| β-ACTIN | CACCATTGGCAATGAGCGGTTC | AGGTCTTTGCGGATGTCCACGT |
| MEX3C | AGAAAGAGCGTCAACACCACC | AAATGGGCTCTTCACCACGAA |

**Table 2. Sequences used for transfection of siRNA**

| **MEX3C** | **siRNA1** | **siRNA2** |
| --- | --- | --- |
| **sense (5’-3’)** | GCGUACAGGAAACUAUAUATT | GCUCUACAGAUUCCUACUUTT |
| **antisense (5’-3’)** | UAUAUAGUUUCCUGUACGCAT | AAGUAGGAAUCUGUAGAGCCA |
