# Supplementary material for: MEX3C as a potential target for hepatocellular carcinoma drug and immunity: combined therapy with Lenvatinib
Source: BMC Cancer. 2023 Oct 12;23:967. doi: 10.1186/s12885-023-11320-4 (PMC10568896; doi:10.1186/s12885-023-11320-4)
Supplement: Supplementary file 2 — Supplementary Material 2 [file 12885_2023_11320_MOESM2_ESM.docx]

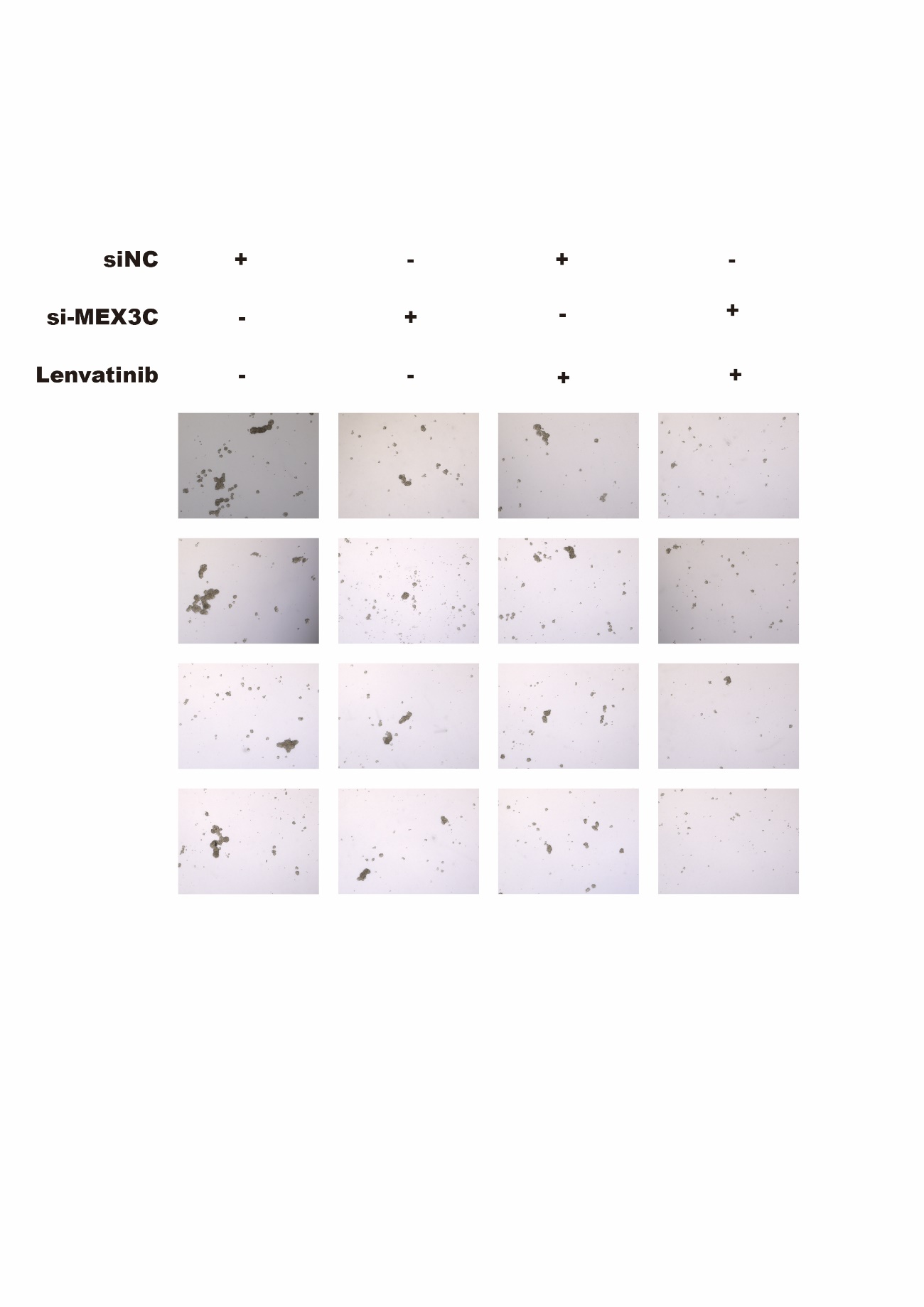


**The supplementary Figure1** **4X mirror field of view in clonogenic sphere formation experiment** The data provided were captured using a 4x microscope. In comparison to treatment with Lenvatinib alone, the knockdown of MEX3C led to a decrease in the stemness and spheroidization ability of Huh7 cells.


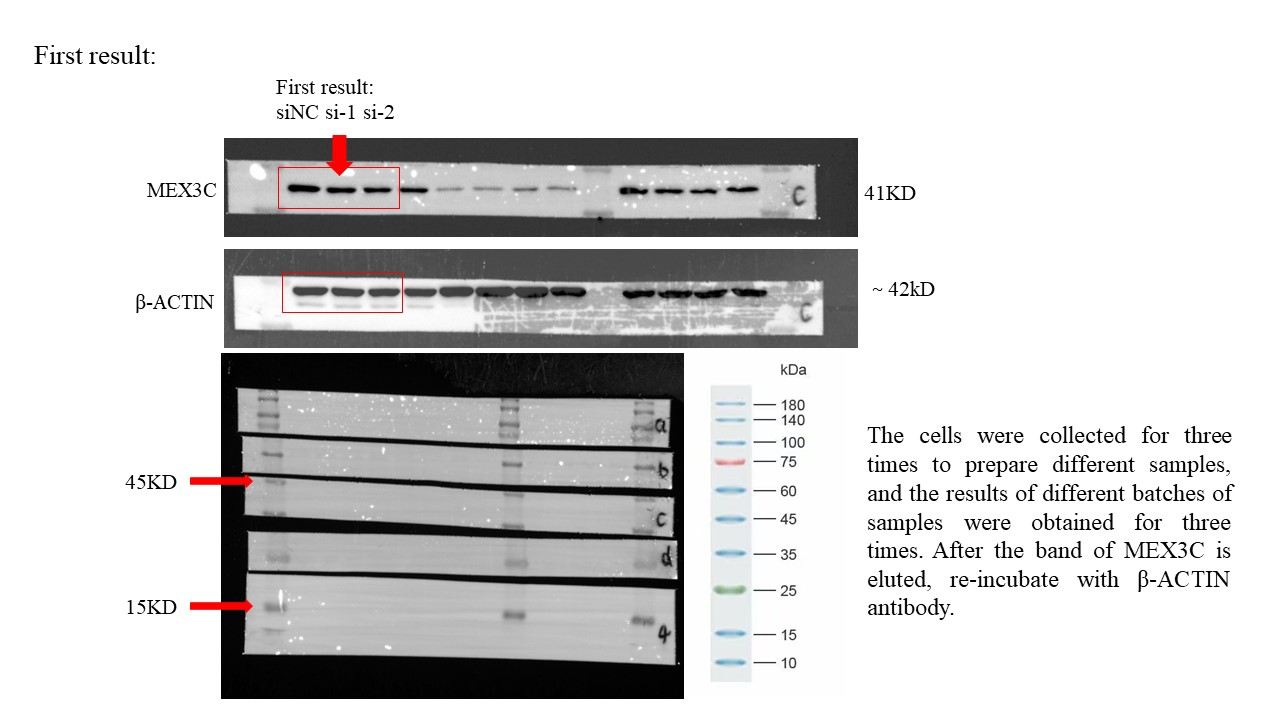


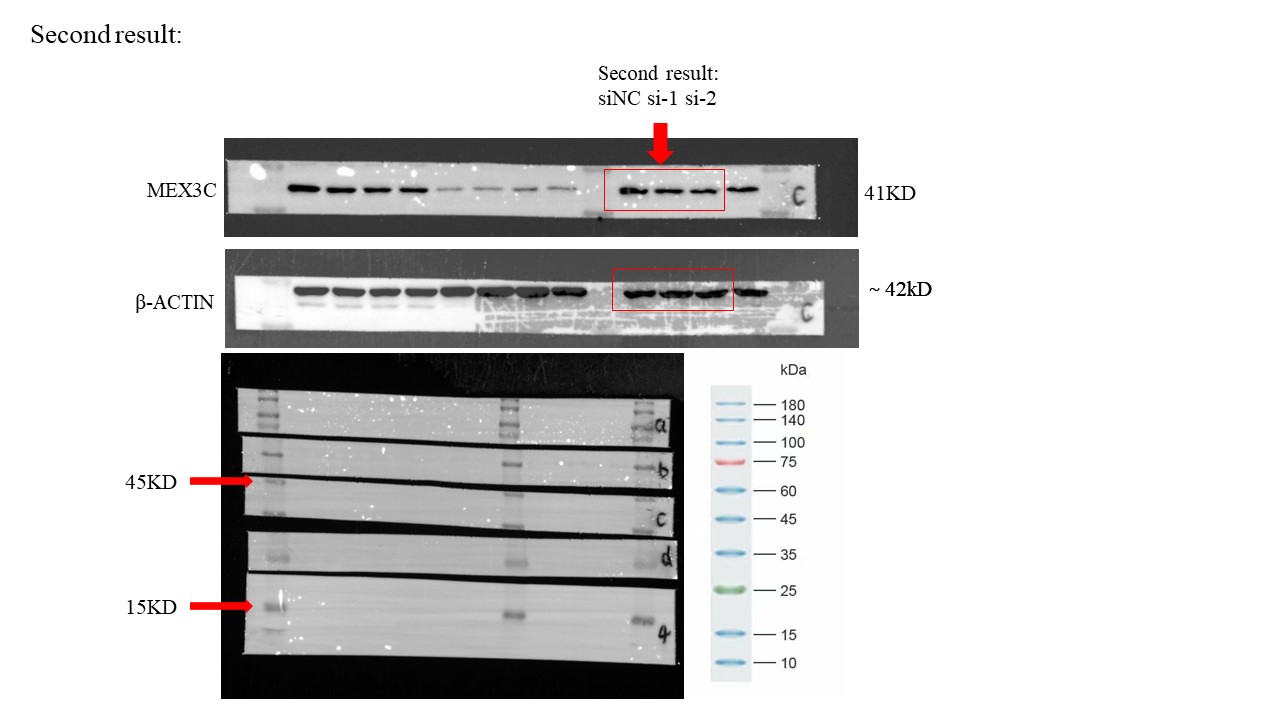


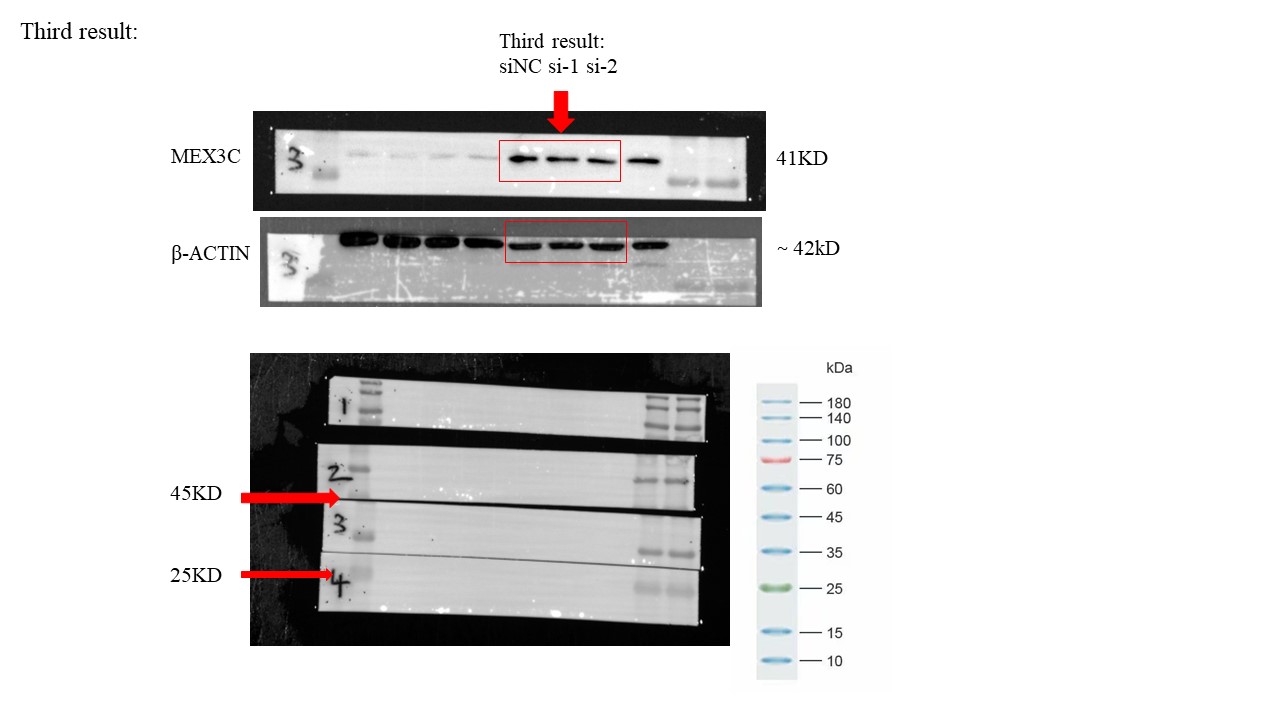


**The supplementary Figure2 WB raw data of MEX3C knockdown** The cells were collected for three times to prepare different samples, and the results of different batches of samples were obtained for three times. After the band of MEX3C is eluted, re-incubate with β-ACTIN antibody.
